# Temporal integration of mitochondrial stress signals by the PINK1:Parkin pathway

**DOI:** 10.1186/s12860-019-0220-5

**Published:** 2019-08-14

**Authors:** J. Logan Bowling, Mary Catherine Skolfield, Wesley A. Riley, Andrew P. Nolin, Larissa C. Wolf, David E. Nelson

**Affiliations:** Department of Biology, Middle Tennessee State University, Murfreesboro, TN 37132 USA

**Keywords:** Cell signalling, Mitophagy, Parkin, PTEN-induced putative kinase 1 (PINK1), Ubiquitin

## Abstract

**Background:**

The PINK1:Parkin pathway regulates the autophagic removal of damaged and dysfunctional mitochondria. While the response of this pathway to complete loss of ΔΨm, as caused by high concentrations of mitochondrial ionophores, has been well characterized, it remains unclear how the pathway makes coherent decisions about whether to keep or purge mitochondria in situations where ΔΨm is only partially lost or exhibits fluctuations, as has been observed in response to certain types of cellular stress.

**Results:**

To investigate the responses of the PINK1:Parkin pathway to mitochondrial insults of different magnitude and duration, controlled titration of the mitochondrial protonophore, CCCP, was used to manipulate ΔΨm in live cells, and the dynamics of PINK1 and Parkin recruitment was measured by fluorescence microscopy. In contrast to the stable accumulation of PINK1 and Parkin seen at completely depolarized mitochondria, partial depolarization produced a transient pulse of PINK1 stabilization and rapid loss, which was driven by small fluctuations in ΔΨm. As the rate of Parkin dissociation from the mitochondria and phospho-polyubiquitin chain removal was comparatively slow, repetitive pulses of PINK1 were able to drive a slow step-wise accumulation of Parkin and phospho-polyubiquitin leading to deferred mitophagy.

**Conclusion:**

These data suggest that the PINK1:Parkin mitophagy pathway is able to exhibit distinct dynamic responses to complete and partial mitochondrial depolarization. In this way, the pathway is able to differentiate between irretrievably damaged mitochondria and those showing signs of dysfunction, promoting either rapid or delayed autophagy, respectively.

**Electronic supplementary material:**

The online version of this article (10.1186/s12860-019-0220-5) contains supplementary material, which is available to authorized users.

## Background

The mitochondria are vitally important, multifunctional organelles, responsible for ATP production, the regulation of apoptosis, and other cell type-specific processes. A cell’s mitochondrial network is capable of remodelling itself – expanding or diminishing in size to meet the metabolic demands of the cell – or ‘pruning’ and destroying dysfunctional regions of the network by mitophagy to maintain its integrity. While several pathways exist that regulate the autophagic removal of mitochondria from the cell [1–4], the PTEN-induced putative kinase 1 (PINK1):Parkin pathway is perhaps the most prominent and most closely associated with the destruction of mitochondria in response to stress or loss of mitochondrial membrane potential (ΔΨm) in normal, somatic cells [2, 5]. Indeed, mutation of either the genes encoding the PINK1 and Parkin proteins (*PINK1* and *PRKN*, respectively) can result in the accumulation of dysfunctional mitochondria in animal and cell models [6] and is associated with juvenile onset autosomal recessive forms of Parkinson’s disease (PD; [7–9]).

The PINK1 protein itself is a highly labile, nuclear-encoded, mitochondrially-targeted kinase that essentially acts as a sensor of mitochondrial membrane potential (ΔΨm). In normal, polarized mitochondria, PINK1 is translocated into the mitochondria by the translocase of the outer mitochondrial membrane: translocase of the inner membrane (TOM:TIM) complex. There, the amino-terminus of PINK1 undergoes two rounds of proteolytic processing; the mitochondrial targeting sequence is removed by matrix-localized proteases and then a larger portion of the protein is removed by PGAM5-associated rhomboid-like protease (PARL) [10–12]. The mature 52 kDa PINK1 protein is then retrotranslocated to the cytosol side of the outer mitochondrial membrane (OMM), ubiquitinated, and rapidly degraded by the 26S proteasomes, thereby maintaining a low basal concentration of PINK1. However, as the operation of TIM23 – the component of the TOM:TIM complex that interacts directly with the N-terminus of PINK1 [13] – is ΔΨm dependent, at depolarized regions of a cell’s mitochondrial network, PINK1 accumulates at the OMM. Here, it is able to activate Parkin directly via phosphorylation at Ser65 within the N-terminal Ubl domain and indirectly via production of phospho-ubiquitin moieties [14–17]. PINK1 and Parkin then operate together to assemble phospho-polyubiquitin (ppUb) chains on OMM substrates with ppUb serving as docking sites for Parkin [18, 19], amplifying the response [20]. These ppUb chains are also bound by autophagy receptors, including optineurin (OPTN) and nuclear dot protein 52 (NDP52) [21, 22]. These bind both ubiquitin and microtubule-associated protein light chain 3 (LC3), promoting the sequestration of ppUb-labelled mitochondria within autophagosomes to be degraded later after fusion with lysosomes.

The relationship between PINK1 and Parkin, and the phospho-polyubiqutin chains that the two proteins synthesize, together constitute two interlinked coherent feed-forward loops (FFLs) – the Parkin activation FFL and phospho-polyubiquitin FFL – followed by a positive feedback loop [19, 20, 23]. Coherent FFLs consist of two activators that coregulate an output, with one of the activators capable of activating the other [24]. Within the context of the PINK1:Parkin pathway, it is likely that these FFLs create an initial delay in the accumulation of phospho-polyubiqutin chains at the OMM, but once a critical concentration of these chains has been reached, the positive feedback produced by the recruitment of Parkin to the chains – their own product – acts as an amplifier, increasing the rate of their production. It is currently hypothesized that this arrangement of feedback loops enables the PINK1:Parkin pathway to essentially filter out transient mitochondrial stress signals, only committing mitochondria to mitophagy if they exhibit a persistent loss of ΔΨm [20]. This has been tested in vitro in various studies using reversible inhibitors of oxidative phosphorylation, such as carbonyl cyanide *m*–chlorophenyl hydrazone (CCCP), applied at high concentrations to completely depolarize the mitochondria for different periods of time [25, 26]. While these studies have been incredibly revealing, they may not accurately recapitulate the characteristics of mitochondrial stresses experienced in vivo, which are likely to be complex, and the changes in ΔΨm that they produce are likely to be entangled with natural or functionally important variation in mitochondrial activity as part of a cell’s normal operations [27–29]. It therefore remains an open question as to how the PINK1:Parkin pathway successfully interprets mitochondrial stress signals, especially in situations where ΔΨm is only partially lost or where the cell experiences intermittent exposure to mitochondrial stresses.

In this study, we use a live cell imaging approach to investigate how the PINK1:Parkin pathway processes different stress signals. By monitoring ΔΨm and the expression and localization of PINK1 and Parkin proteins in individual cells, we show that at intermediate levels of mitochondrial stress, where ΔΨm is only partially lost, the temporal dynamics of PINK1 and Parkin differ markedly. While a complete and sustained loss of ΔΨm induced by high concentrations of CCCP leads to stable association of both PINK1 and Parkin with the mitochondria, a partial loss of ΔΨm produces transient pulses of PINK1 accumulation in cells. Loss of mitochondrial PINK1 at the conclusion of these pulses is preceded by a small, partial recovery in ΔΨm even when cells are exposed to constant concentrations of CCCP. Furthermore, when cells are exposed to repeated rounds of mitochondrial stress, occurring on a timescale of minutes or hours, PINK1 is not influenced by prior depolarization events and responds to each event independently, quickly returning to basal levels upon partial or complete recovery of ΔΨm. However, as PINK1 and Parkin operate on different timescales, with Parkin dissociating relatively slowly from phospho-polyubiquitin chains on the OMM after repolarization, repeated rounds of mitochondrial depolarization can cause a step-like accumulation of mitochondrial Parkin if mitochondrial depolarization events are sufficiently frequent or if the mitochondria remain in a persistent state of partial depolarization. In this way, Parkin and phospho-polyubiqitin at the OMM can act as a marker of prior mitochondrial stress, triggering mitophagy if ΔΨm is partially lost for extended periods of time or if fluctuations in ΔΨm occur at high frequency but filter out or “ignore” infrequent, stochastic fluctuations in ΔΨm.

## Results

### Titration of CCCP to generate states of partial and complete loss of mitochondrial membrane potential

**Fig. 1:**
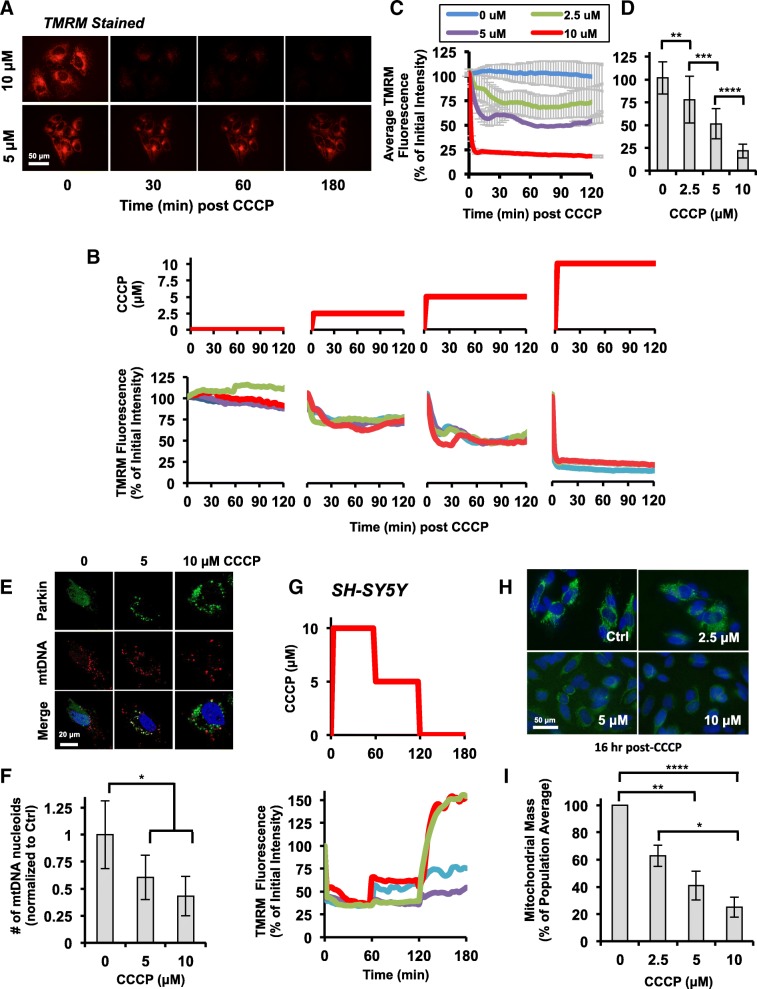
Complete and partial depolarization of mitochondria through titration of CCCP. **a** fluorescent timelapse microscopy images of TMRM-stained HeLa cells at the indicated times post 10 or 5 μM CCCP treatment. **b** CCCP treatment regimes (top) and corresponding single-cell trajectories of TMRM fluorescence in HeLa cells (bottom). Data from 4 representative cells is shown for each treatment. Trajectories for each cell are represented in a different colour. **c** population average trajectories of TMRM fluorescence for cells depicted in (**b**). **d** average TMRM staining for cells 120 min post-CCCP treatment. Data is presented as an average of 3 biological repeats with a minimum of 18 cells per condition. For (**c**) and (**d**), error is represented as the standard error (S.E.). **e** *+* **f** HeLa cells expressing EYFP-Parkin (green) were incubated with the indicated concentrations of CCCP for 24 h, fixed, and stained with DAPI (blue) and antibodies to detect mtDNA nucleoids (red), then imaged by confocal microscopy. Representative images are shown in (**e**). The average number of mtDNA puncta were quantified for 20 cells per condition and presented in (**f**) normalized to the number of puncta in control cells. **g** CCCP treatment regimes (top) and corresponding single-cell trajectories of TMRM fluorescence in SH-SY5Y cells (bottom). Data from 4 representative cells is shown. **h** fluorescence images of SH-SY5Y cells stained with MitoTraker Green FM (green) and Hoechst 33342 16 h post-CCCP. **i**, quantification of average mitochondrial mass for cells exposed for 16 h to the indicated concentrations of CCCP. Staining intensity was normalized to the population average for the control cells and presented as the average of the independent repeats. Error is represented as the S.E.. Data is from a minimum of 131 cells per condition across 5 biological repeats. For (**d**, **f**, and **i**) statistical differences in TMRM fluorescence between treatment conditions was appraised by one-way ANOVA and Tukey’s multiple comparisons follow-up test. Statistical significance is indicated as follows: *, *p* < 0.05; **, *p* < 0.01; ***, *p* < 0.001; and ****, *p* < 0.0001. Scale bars represent 50 μm in (**a** *+* **g**) and 20 μm in (**e**)

In order to investigate how the PINK1:Parkin mitophagy pathway responds to complex stress signals – defined here as transient or continuous, complete or partial loss of mitochondrial membrane potential – we utilized the reversible inhibitor of oxidative phosphorylation, CCCP. Low (2.5 μM), medium (5 μM), and high doses (10 μM) of CCCP were selected based on published data using isolated mitochondria showing a dose-dependent reduction in Tim23-dependent protein transport into the mitochondria across this concentration range [30]. In live TMRM-stained HeLa cells, incubation with these doses of CCCP was found to cause a minor, moderate, and complete loss of ΔΨm, respectively (Fig. 1a-d). At the level of individual cells, small, asynchronous fluctuations in ΔΨm in cells incubated with 2.5 and 5 μM CCCP were observed (Fig. 1b), but at the population level, the different levels of ΔΨm produced by the selected doses of CCCP were distinct (Fig. 1c and d). In order to test whether a partial loss of ΔΨm could induce mitophagy, EYFP-Parkin expressing HeLa cells were incubated with 0, 5, or 10 μM CCCP for 24 h and immunofluorescently stained with anti-DNA antibodies to detect mtDNA nucleoids. We observed a ~ 40% and ~ 57% reduction in the number of mtDNA puncta in 5 and 10 μM CCCP treated cells, respectively, when compared to vehicle-treated controls (Fig. 1e-f), indicating that even a moderate loss of ΔΨm can stimulate mitophagy and the degradation of mitochondria.

To determine whether cells expressing endogenous Parkin would exhibit a similar response, we first tested whether different levels of ΔΨm could be achieved and reversed in the dopaminergic, neuroblastoma-derived cell line, SH-SY5Y, by titrating CCCP into the cells followed by wash-out (Fig. 1g). As very similar effects to those observed in HeLa cells were achieved we subsequently tested whether a sustained complete or partial loss of ΔΨm could also stimulate a loss of mitochondrial mass in SH-SY5Y cells. This was achieved by staining cells with the ΔΨm-independent mitochondrial dye, MitoTracker Green FM, after incubation in low, medium, and high doses of CCCP for 16 h. Consistent with our observations in Parkin-expressing HeLa cells, we found that SH-SY5Y cells exhibited a dose-dependent response to CCCP with even medium doses producing a statistically significant reduction in staining, indicating that even a partial loss of mitochondrial membrane potential could stimulate mitophagy (Fig. 1h and i).

### Partial loss of ΔΨm produces transient pulses of mitochondrial PINK1

**Fig. 2:**
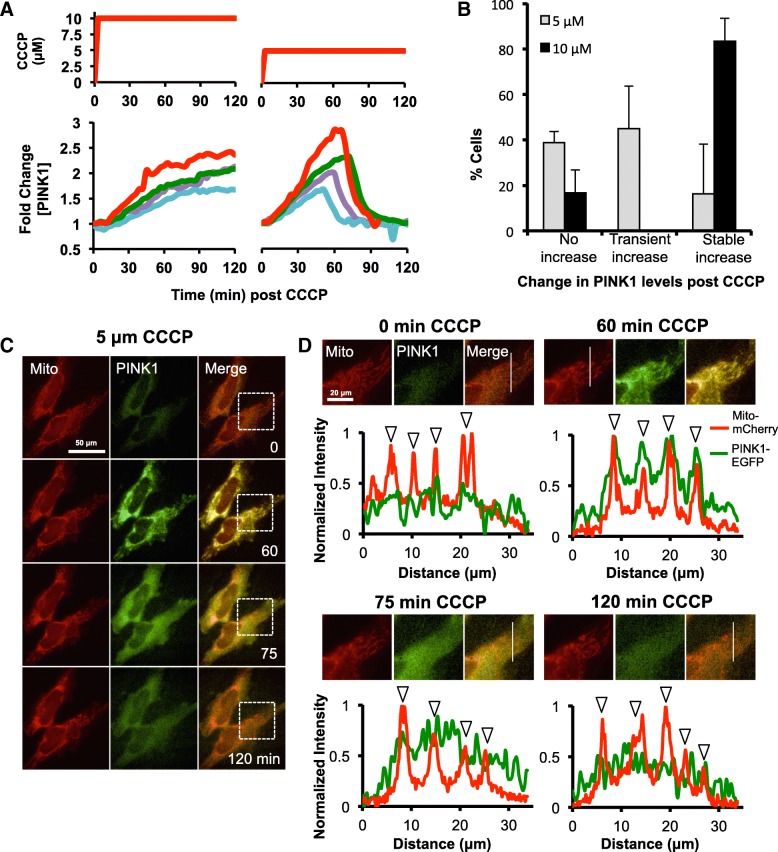
Partial mitochondrial depolarization produces a transient PINK1 response. **a** fluorescence timelapse microscope of HeLa cells expressing with PINK1-EGFP and mito-mCherry using the indicated CCCP treatment regimes (top). Corresponding single-cell trajectories of PINK1-EGFP fluorescence from 4 representative cells is shown for each treatment (bottom). **b** for the experiment described in (**b**), for each treatment group, cells were categorized as “no increase”, if no stabilization of PINK1-EGFP was observed post-CCCP, “transient increase” for cells where PINK1-EGFP increased post-CCCP but returned to basal levels within 2 h, and “stable increase” for cells showing a stable increase in PINK1-EGFP levels. Data is from a minimum of 3 biological repeats with ≥71 cells per condition. Error is represented as the S.E. **c** timelapse fluorescence images of HeLa cells expressing with PINK1-EGFP (green) and mito-mCherry (red) incubated for the indicted times with 5 μM CCCP. Scale bar represents 50 μm. **d** zoomed images of areas represented by the dashed boxes in (**c**). Scale bar represents 20 μm. Pixel intensities for line scans (position marked with thin white line) are presented below. Arrowheads indicate where line scan crosses mitochondria

**Fig. 3:**
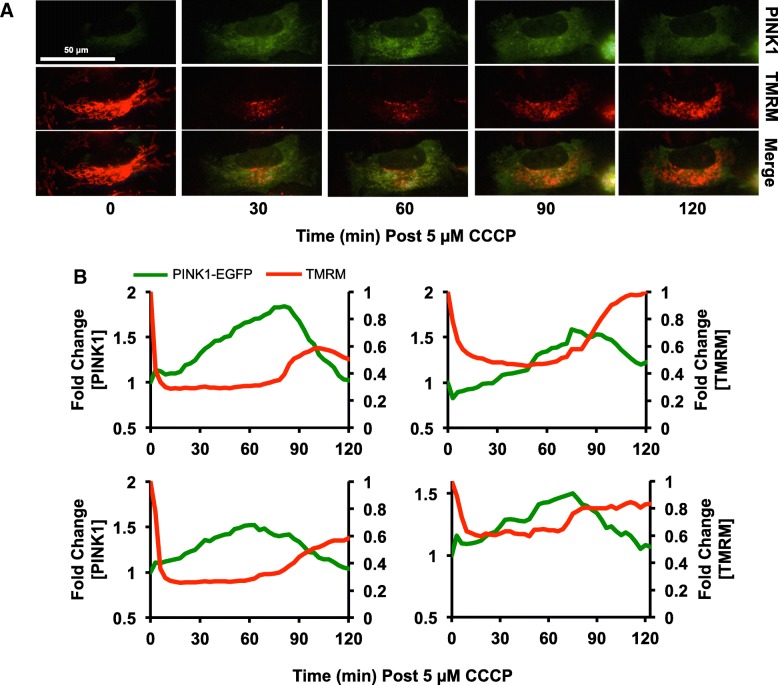
Fluctuations in mitochondrial membrane potential drive transient PINK1 stabilization in cells incubated with low doses of CCCP. **a** time-lapse images of PINK1-EGFP (green) and TMRM (red) fluorescence in a HeLa cell incubated with 5 μM CCCP. Partial recovery of mitochondrial membrane polarization at t = 90 min (as indicated by increased TMRM fluorescence) is followed by a decrease in PINK1-EGFP levels. **b** quantification of PINK1-EGFP and TMRM fluorescence in single live HeLa cells incubated in 5 μM CCCP. In each of the 4 representative cells, increased TMRM fluorescence was associated with rapid loss of PINK1-EGFP. Scale bar represents 50 μm

Many stress-responsive signalling pathways, including NF-κB and p53, produce distinct dynamic behaviours in response to different levels of stimulus in order to provide an appropriate, measured response [31–33]. As we saw a dose-dependent effect of ΔΨm reduction on mitochondrial mass, we hypothesized that the PINK1:Parkin pathway may also exhibit different behaviours in cells where ΔΨm is partially or fully lost. In order to investigate this in live cells, we produced plasmid constructs to express human PINK1-EGFP fluorescent fusion proteins. A C-terminal tagging strategy was used to avoid potentially interfering with the function of the PINK1 N-terminal MTS, which may affect protein trafficking and activity [34, 35]. Having verified that the full-length fusion protein was stabilized after mitochondrial depolarization by western blotting (Additional file 1: Figure S1), we performed live cell imaging on HeLa cells co-expressing PINK1-EGFP and the mitochondrial marker mito-mCherry, incubated with medium and high doses of CCCP (Fig. 2a-d). Consistent with published data, in cells incubated with 10 μM CCCP, PINK1-EGFP fluorescence steadily increased over the 2 h period of observation, colocalizing with the mitochondria (Fig. 2a; [36]). However, in almost 50% of the cells incubated with 5 μM CCCP, PINK1-EGFP accumulation was transient (Fig. 2a and b), rising with similar kinetics to cells incubated with 10 μM before rapidly dissociating and decreasing in levels (Fig. 2d). Interestingly, the loss of PINK1-EGFP fluorescence was asynchronous between cells in the same experiment. Noticing that TMRM fluorescence, and therefore ΔΨm, showed small, random fluctuations in cells treated with medium doses of CCCP (Fig. 1b), we speculated that these could potentially cause TIM23 to briefly switch from the inactive to the active state, leading to rapid internalization and subsequent degradation PINK1-EGFP. To examine this possibility, we monitored both TMRM fluorescence and PINK1-EGFP levels in single cells incubated with 5 μM CCCP and found that PINK1-EGFP loss was concomitant with small increases in ΔΨm (Fig. 3a and b).

### Partial recovery of ΔΨm is sufficient to stimulate loss of mitochondrial PINK1

**Fig. 4:**
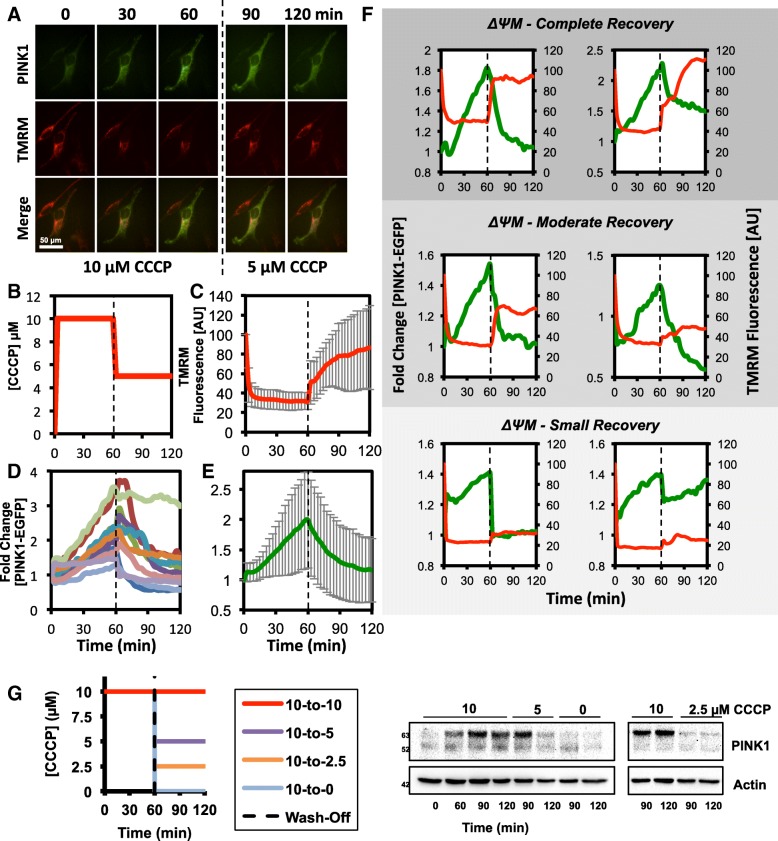
Partial recovery of mitochondrial membrane potential is sufficient to induce PINK1 dissociation from the mitochondria. **a** fluorescence microscopy images of PINK1-EGFP (green) and TMRM (red) fluorescence in HeLa cells maintained in a constant concentration of TMRM (10 nM) and treated with 10 μM CCCP for 60 min to completely depolarize the mitochondria. The mitochondria were then partially repolarized by decreasing the CCCP concentration to 5 μM for the following 60 min, as depicted in the schematic (**b**). The scale bar in (**a**) represents 50 μm. **c***-***e** quantification of (**c**) average TMRM fluorescence in single cells (**d**) PINK1-EGFP fluorescence in 10 representative single cells and (**e**) the population average for PINK1-EGFP fluorescence. **f** For the experiment depicted in (**a**-**e**), the PINK1-EGFP responses of individual cells were separated into categories based on the magnitude of ΔΨM recovery (complete, moderate, or small) after CCCP concentration was decreased from 10 μM to 5 μM. The experiment depicted in (**a**-**f**) was performed as 3 discrete biological repeats with data gathered from a total of 24 individual cells. Error is represented as the standard deviation. **g** HeLa cells were treated with 10 μM CCCP for 60 min and then the mitochondria were partially or fully repolarized by decreasing the concentration of CCCP to 5, 2.5, or 0 μM or they were left in 10 μM CCCP as a control as depicted in the schematic (left). Endogenous PINK1 and actin levels were determined at the indicated timepoints during the treatment regime by western blotting (right)

Complete recovery of ΔΨm after cells have been incubated with high concentrations of CCCP has previously been shown to stimulate rapid loss of mitochondrial PINK1 in population-based immunoblotting assays [36]. However, our data suggests that even a partial recovery of ΔΨm is sufficient to stimulate a similarly rapid return of PINK1 to basal levels in live cells. To explicitly test this, changes in PINK1-EGFP levels and TMRM fluorescence were monitored in live cells when CCCP concentration was reduced from 10 to 5 μM to cause a synchronous partial recovery in ΔΨm (Fig. 4a-g). As expected, reducing CCCP concentration caused a partial recovery of ΔΨm (Fig. 4a-c), and, in most cells, this was accompanied by a rapid reduction in PINK1-EGFP levels (Fig. 4a, d and e). Based on TMRM fluorescence measurements, the change in ΔΨm after reduction of CCCP concentration was rapid, generally reaching a new steady-state within minutes (Fig. 4c and f). While the magnitude of ΔΨm recovery differed markedly between cells within the same population, a concomitant loss of PINK1-EGFP levels was observed in most cells, even those showing a moderate or small recovery in TMRM fluorescence (Fig. 4f).

To examine whether the dynamics of exogenous PINK1-EGFP was consistent with endogenous PINK1 proteins, the levels of endogenous PINK1 were measured by western blotting in cells after partial or complete repolarization by incubation with 5, 2.5, or 0 μM CCCP after 1 h in 10 μM CCCP. In agreement with the live cell experiments, unprocessed, full-length PINK1 proteins decreased to near-basal levels after partial repolarization, although this appeared to occur more rapidly at lower CCCP concentrations (Fig. 4g). Consistent with published data, cells exhibited a rapid and complete loss of full-length PINK1 when CCCP was removed entirely [25, 36].

### The rate of Parkin loss from the mitochondria is slower than PINK1 after partial or complete recovery of ΔΨm

**Fig. 5:**
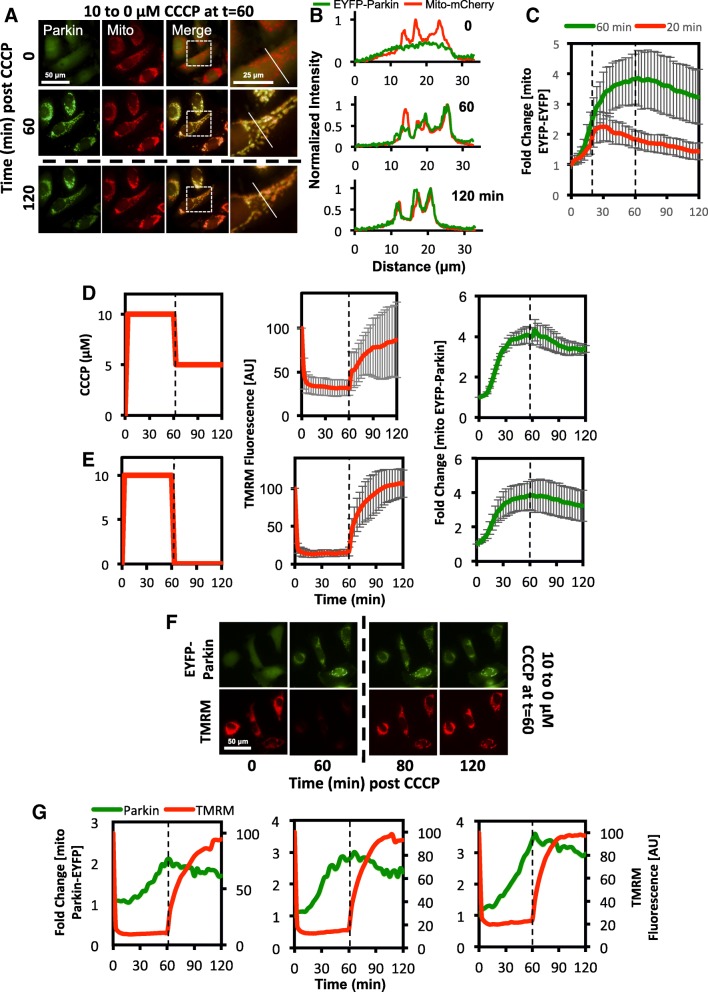
Dissociation of Parkin from the mitochondria occurs at a slower rate than PINK1. **a**-**c** HeLa cells expressing EYFP-Parkin and mito-mCherry were imaged by live cell fluorescence microscopy in the presence of 10 μM CCCP, which was washed out after either 20 or 60 min. Timecourse images of cells exposed to 10 μM CCCP for 60 min are shown in (**a**). In these cells, EYFP-Parkin (green) colocalizes with the mitochondrial marker (mito-mCherry) within 60 min after initial CCCP exposure and remains colocalized for at least 60 min after CCCP removal (120 min timepoint). Thin white lines in the zoomed images mark the trajectory of the line scan measurement and the intensity values for EYFP-Parkin and mito-mCherry fluorescence are shown in (**b**). Quatification of the change in EYFP-Parkin levels at the mitochondria are shown as (**c**). Trajectories represent average fold change in mitochondrial EYFP-Parkin from 3 biological repeats with a minimum of 44 cells per condition. **d**-**g** HeLa cells expressing EYFP-Parkin and stained with 10 nM TMRM were imaged by live cell microscopy in the presence of 10 μM CCCP. After 60 min, the concentration of CCCP was reduced to (**d**) 5 μM or (**e**-**g**) 0 μM to either partially or fully restore mitochondrial membrane potential for the final 60 min of the experiment. For (**d** *+* **e**) a representation of the CCCP treatment regime (left), quantification of average TMRM fluorescence (center), and average fold change of EYFP-Parkin localized to mitochondria (right) is shown. Error is represented as the S.E. Representative fluorescence microscopy images and single cell trajectories for mitochondrial EYFP-Parkin and TMRM levels for cells exposed to 10 μM CCCP for 60 min followed by 0 μM CCCP for the following 60 min is shown as (**f**) and (**g**), respectively

As we showed that even a small increase in ΔΨm can stimulate a rapid loss of PINK1, we hypothesized that this should also stimulate Parkin dissociation from the OMM. However, in live cell imaging experiments, we found that EYFP-Parkin dissociated from mitochondria far slower than PINK1 on complete removal of CCCP 60 min post-treatment with EYFP-Parkin remaining largely colocalized with the mitochondrial marker, mito-mCherry in HeLa cells even 1 h after CCCP washout (Fig. 5a and b). Furthermore, the rate of EYFP-Parkin dissociation was similar when CCCP was removed at 20 or 60 min post-treatment (Fig. 5c). To test whether partial repolarization of the mitochondria was sufficient to stimulate Parkin dissociation from the mitochondria, we decreased the concentration of CCCP from 10 to 5 μM at 60 min post-treatment and measured the fold change in mitochondrial EYFP-Parkin levels by live cell imaging (Fig. 5d). Here, we also observed a slow decrease in mitochondrial EYFP-Parkin, identical to that observed in cells where CCCP was completely removed (Fig. 5d and e). As an additional control, these experiments were repeated in the presence of TMRM to test whether ΔΨm increased after CCCP washout (Fig. 5f and g). These experiments clearly showed that Parkin remained associated with the mitochondria over longer timescales than PINK1, remaining present for many minutes after recovery of ΔΨm (Fig. 5g).

**Fig. 6:**
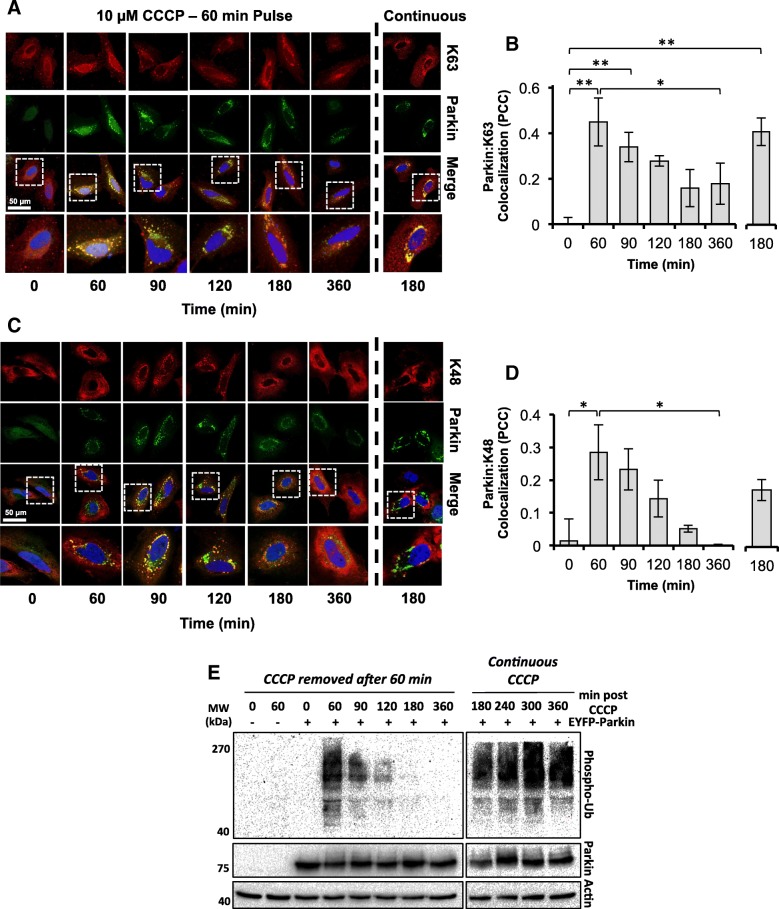
The persistence of phosphorylated poly-ubiquitin at the OMM allows for Parkin retention after repolarization of mitochondria. **a**-**e** HeLa cells expressing EYFP-Parkin (green) were treated with either a 60 min pulse or continuously with 10 μM CCCP, fixed at the indicated timepoints and stained with DAPI (blue) and antibodies to detect (**a**) K63 polyubiquitin or (**c**) K48 polyubiquitin (red) then imaged by confocal microscopy. Scale bars represent 50 μm. For both (**a** *+* **c**), zoomed images of areas represented by the dashed boxes are presented below the ‘merge’ images. The colocalization of EYFP-Parkin with (**b**) K63 or (**d**) K48 was assessed using Pearson’s correlation coefficient (PCC) and graphed as the average PCC score. Data is from 3 biological repeats with a minimum of 56 cells per condition. Error is represented as the S.E. Statistical differences in PCC score were appraised by one-way ANOVA and Tukey’s multiple comparisons follow-up test. Statistical significance is indicated as follows: *, *p* < 0.05; **, *p* < 0.01. *E,* Western blot analysis for phospho-polyubiquitin levels in EYFP-Parkin expressing HeLa cells treated with either a 60 min pulse or continuously with 10 μM CCCP

We speculated that the retention of Parkin could be caused by the persistence of ppUb chains at the OMM after repolarization, which could enable Parkin to remain tethered to the mitochondria independent of PINK1. It is known that Parkin is capable of conjugating K6, K11, K48, and K63 polyubiquitin chains to protein substrates [23], and PINK1 is capable of phosphorylating K48 and K63 chains to produce ppUb [18]. In order to test our hypothesis we performed immunofluorescent staining with pan or linkage-specific anti-ubiquitin antibodies to measure changes in Parkin colocalization with polyubiquitin in HeLa cells after de- and repolarization of mitochondria. Polyubiquitin could be detected at parkin-positive mitochondria by 60 min post-CCCP treatment (Additional file 2: Figure S2). Consistent with our hypothesis, we found that this colocalization was maintained for at least 2 h after CCCP washout (Additional file 2: Figure S2). Similarly, the degree of Parkin colocalization with both K48 and K63 polyubiquitin increased after incubation of the cells with 10 μM CCCP for 60 min and steadily decreased over a 5 h period after CCCP wash-out (Fig. 6a-d). Interestingly, the kinetics of this process differed between the two chain types with Pearson’s correlation coefficient (PCC) values for Parkin:K48 polyubiquitin colocalization decreasing faster than for Parkin:K63. Furthermore, while all parkin-positive mitochondria also stained positive for K63 polyubiquitin chains throughout the experiment (Fig. 6a), within 30 min after CCCP removal (and also by 180 min in continuously treated cells) K48 staining appeared to be completely absent at a subset of parkin-positive mitochondria (Fig. 6c), suggestive of differing roles for K48 and K63 polyubiquitin chains during mitophagy. When the levels of phospho-polyubiquitin in these cells were tested directly by western blotting, phospho-polyubiquitin was detectable but much diminished in whole cell lysates at 90 min post-CCCP wash-out (Fig. 6e), consistent with the findings of the immunofluorescent staining assays.

### The autophagy receptor, OPTN, is also retained at Parkin-positive mitochondrial fragments after repolarization of mitochondria

**Fig. 7:**
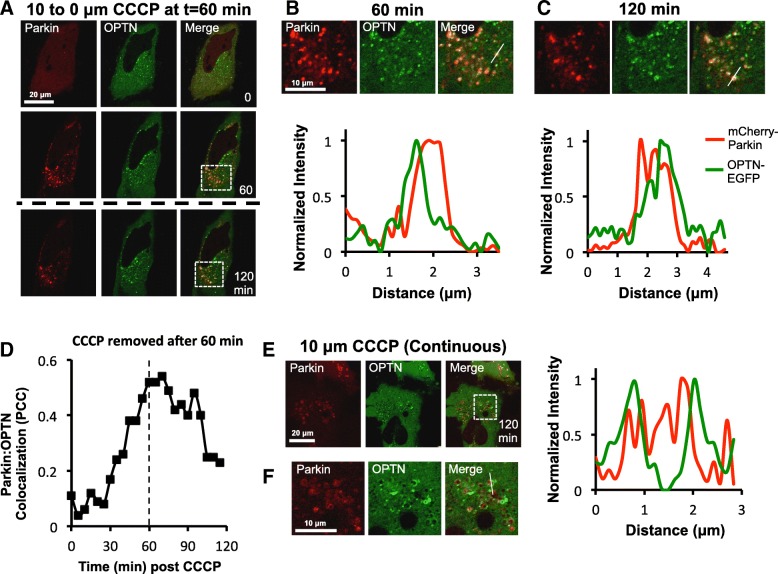
The autophagy receptor, OPTN, is also retained at Parkin-positive mitochondria after repolarization. **a**-**f** HeLa cells expressing mCherry-Parkin and OPTN-EGFP were imaged by live cell microscopy and were either (**a**-**d**) pulsed for 60 min or (**e** *+* **f**) treated continuously with 10 μM CCCP. Timecourse images for cells pulsed for 60 min are depicted in (**a**), together with corresponding zoomed images and line scan pixel intensity measurements of mCherry-Parkin and OPTN-EGFP fluorescence taken at (**b**) 60 min and (**c**) 120 min. Thin white lines mark the trajectory of the line scan measurement in (**b** *+* **c**). OPTN-EGFP (green) puncta are found to be associated with mitochondrial fragments coated with mCherry-Parkin (red) within 60 min after initial CCCP exposure and these remain associated for at least 60 min post-CCCP washout. **d** For the cell depicted in (**a**-**c**), colocalization between mCherry-Parkin and OPTN-EGFP within a ~ 10 × 10 μm region of the cytosol was assed overtime by PCC. **e** Image of cells at 120 min after continuous treatment with CCCP together with (**f**) corresponding zoomed images and line scan pixel intensity measurements of mCherry-Parkin and OPTN-EGFP fluorescence. At this timepoint crescents or rings of OPTN surround EYFP-Parkin-coated mitochondrial fragments

If the extended retention of Parkin after repolarization of mitochondria is due to the slow removal of ppUb, this would suggest that autophagy receptors, such as OPTN, that bind to ppUb will also be retained. In order to test this, mCherry-Parkin and OPTN-EGFP fusion proteins were expressed in HeLa cells. These cells were imaged by live cell microscopy and either pulsed for 60 min or treated continuously with 10 μM CCCP. Prior to CCCP exposure, OPTN-EGFP was spread homogeneously throughout the cytoplasm with scattered puncta visible in most cells (Fig. 7a). Consistent with published data [37], OPTN-EGFP puncta were rapidly recruited to mCherry-Parkin-coated mitochondria and were clearly visible by 60 min (Fig. 7a and b).

Although fewer Parkin-positive mitochondria were observable by 120 min in pulsed cells compared to continuously treated cells (Additional file 3: Figure S3A), OPTN puncta were still associated with fragmented mitochondria at 120 min, 60 min post-CCCP wash-off (Fig. 7a, c and Additional file 3: Figure S3B) with OPTN-EGFP:mCherry-Parkin colocalization slowly diminishing over time (Fig. 7d). By the same time in the continuously treated cells, OPTN-EGFP puncta had spread over the surface of mCherry-Parkin-coated fragmented mitochondria, forming crescents or rings (Fig. 7e and f), suggesting that although OPTN-EGFP could be retained for a short period of time after mitochondrial repolarization, the process of coating mitochondrial fragments with this autophagy adaptor was arrested and does not go to completion. This may prevent mitochondria that exhibit a transient decrease in ΔΨm from being sequestered within autophagosomes.

### The PINK1 response to loss of ΔΨm is stereotyped and is not influenced by prior depolarization events

**Fig. 8:**
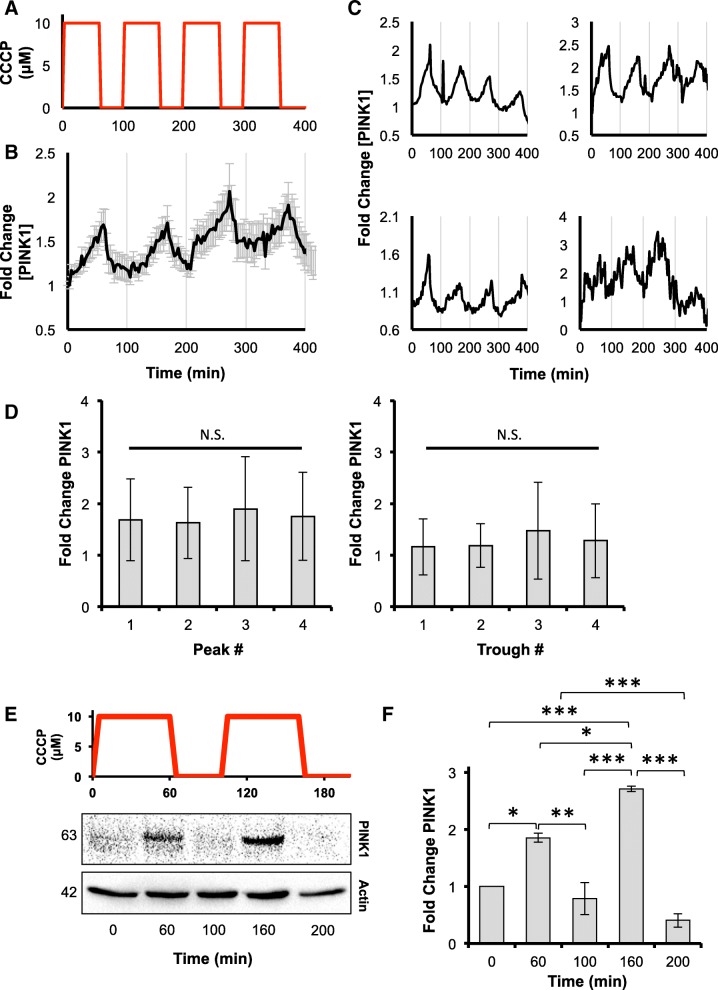
The PINK1 response to cycles of de- and repolarization of the mitochondria is stereotyped. **a**-**d** HeLa cells expressing PINK1-EGFP were exposed to 60 min pulses of 10 μM CCCP every 100 min, as depicted in the schematic (**a**), and imaged by live cell fluorescence microscopy. **b** population average trajectories of PINK1-EGFP fluorescence based on 3 biological repeats and a total of 19 cells. Error is represented as the S.E. **c** trajectories of PINK1-EGFP fluorescence for 4 representative cells. **d**, peak and trough analysis of PINK1-EGFP levels. Error is represented as the S.E. Statistical significance was determined by one-way ANOVA. **e** HeLa cells were treated with 60 min pulses of 10 μM CCCP every 100 min, as represented in the schematic, harvested at the indicated time points and immunoblotted for PINK1 and actin as a loading control. **f** PINK1 levels at each timepoint were quantified by densitometry and normalized to actin. Statistical differences in PINK1 levels between samples were appraised by one-way ANOVA and Tukey’s multiple comparisons follow-up test. Statistical significance is indicated as follows: *, *p* < 0.05; **, *p* < 0.01; ***, *p* < 0.001. Data is from 3 biological repeats

As our data suggested that PINK1 and Parkin are lost from the mitochondria at different rates after recovery of ΔΨm, we hypothesized that fluctuations in ΔΨm could produce discrete pulses of mitochondrial PINK1 activity that would drive the slow accumulation of phospho-polyubiquitin, potentially triggering mitophagy even in cells that did not exhibit a persistent loss of ΔΨm. To investigate this, PINK1-EGFP fluorescence was measured in HeLa cells exposed to 60-min pulses of 10 μM CCCP every 100 min by live cell microscopy (Fig. 8a). As expected, this treatment regime produced discrete pulses of PINK1-EGFP, detectable at the population level (Fig. 8b), and in individual cells (Fig. 8c). Notably, PINK1-EGFP fluorescence dropped to basal levels between each pulse, and there was no significant difference in the amplitude of the peaks (Fig. 8d), indicating that the prior history of the cell did not influence the magnitude of the PINK1 response to successive CCCP treatments. Similar results were also obtained for endogenous PINK1 in HeLa cells although the quantity of full-length PINK1 accumulating after the second CCCP pulse was slightly greater (Fig. 8e and f). Together, these data suggest that the mitophagy pathway – at least at the PINK1 level – does not exhibit a refractory state where a prior response inhibits or blocks a response to subsequent stimulus, as has been observed in other stress response pathways [38].

### Pulses of PINK1 activity can promote an incremental accumulation of mitochondrial Parkin and phospho-polyubiquitin

**Fig. 9:**
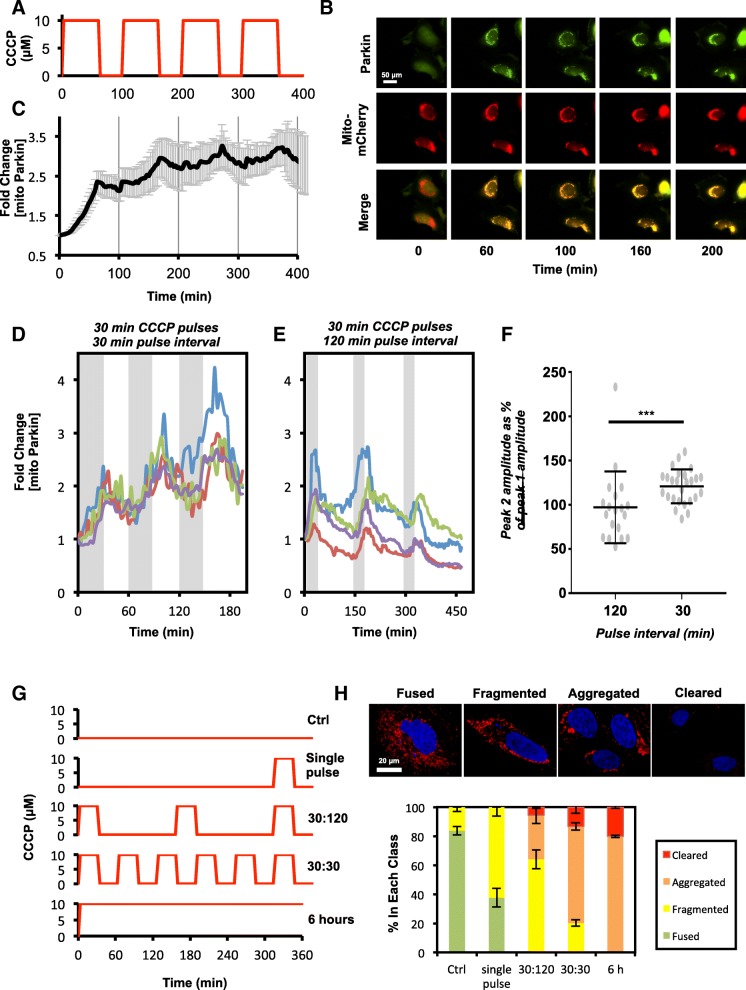
Parkin levels at the mitochondria build in response to repetitive cycles of de- and repolarization. **a**-**c** HeLa cells expressing EYFP-Parkin and mito-mCherry were exposed to 60 min pulses of 10 μM CCCP every 100 min, as depicted in the schematic (**a**), and imaged by live cell fluorescence microscopy. **b** images of representative cells from the experiment. Scale bar represent 50 μm. **c** population average trajectories of the fold change in mitochondrial EYFP-Parkin fluorescence based on 3 biological repeats and a total of 23 cells. Error is represented as the S.E. **d**-**f** the experiment depicted in (**a**-**c**) was repeated using 30 min pulses of 10 μM CCCP separated by (**d**) 30 min or (**e**) 120 min and the trajectories of the fold change in mitochondrial EYFP-Parkin fluorescence for 4 representative cells is shown. The grey bars represent CCCP treatment times. **f** the peak amplitude of mitochondrial Parkin after exposure to the second pulse of CCCP is expressed as a percentage of the amplitude of peak mitochondrial Parkin levels after the first pulse of CCCP. Data is from a minimum of 30 cells per condition across at least 4 biological repeats. Statistical differences in peak amplitude was appraised using a Mann-Whitney test and significance is indicated as follows: ***, *p* < 0.001. **g**-**h**, EYFP-Parkin-expressing HeLa cells were exposed to CCCP, as depicted in the schematic (**g**), fixed and stained with DAPI (blue) and anti-TOM20 antibodies (red), and imaged by confocal microscopy. **h** The percentage of Parkin-expressing cells showing fused, fragmented, aggregated, or cleared mitochondria was determined based on 3 biological repeats with ≥70 cells/condition. Error is represented as the S.E. Representative images for each of the scored groups are shown above the graph. Scale bar represents 20 μm

In order to determine how pulses of PINK1 activity affect accumulation of Parkin at the mitochondria, we employed the same treatment regime used in the prior PINK1 live cell imaging experiments in EYFP-Parkin-expressing HeLa cells (Fig. 9a and b). At the population level, we observed that the first round of CCCP treatment induced a large increase in mitochondrial EYFP-Parkin, and, although small decreases in mitochondrial EYFP-Parkin were observed during the intervals between CCCP treatments, mitochondrial EYFP-Parkin did not drop back to basal levels throughout the duration of the experiment (Fig. 9c). In this regard, the pulses of mitochondrial PINK1 induced by each round of CCCP treatment served to maintain mitochondrial Parkin levels with the relatively slow rate of phospho-polyubiquitin chain loss at the OMM, preventing mitochondrial Parkin dropping back to basal levels between each pulse.

As our previous experiments indicated that mitochondrial EYFP-Parkin levels reach a plateau, becoming near-saturating within 60 min of CCCP treatment (Fig. 5c), we next examined the effects of shorter CCCP pulses on the dynamics of mitochondrial EYFP-Parkin recruitment. The duration of each pulse was reduced to 30 min, which is sufficient to induce PINK1 and Parkin accumulation at the OMM but not long enough for stable recruitment of autophagy receptors or LC3, which takes approximately 35–60 min [37, 39]. For the initial experiment, the pulses were separated by 30 min intervals. Here, we saw a clear increase in the quantity of mitochondrial EYFP-Parkin after successive CCCP pulses (Fig. 9d). However, when the interval between successive pulses was increased to 120 min – the time required for complete removal of phospho-polyubiquitin induced by a prior pulse (Fig. 6d) – the level of mitochondrial Parkin accumulation after each pulse appeared identical in individual cells (Fig. 9e). This was confirmed by statistical analysis, showing that the relative amplitude of the second peak (the maximum level of mitochondrial EYFP-Parkin achieved during each pulse) compared to the first was greater in cells where the pulse interval was only 30 min (Fig. 9f). In order to determine whether this result was meaningful at the level of mitophagy, we exposed cells to the same cycles of CCCP treatment and removal (30 min on:30 min off or 30 min on:120 min off), continuous treatment with CCCP, or a single 30 min pulse of CCCP during a 6 h-long experiment (Fig. 9g). As expected, a greater number of cells exhibited aggregated or cleared mitochondria when cells were pulsed with CCCP at 30 min intervals (80%) than at 120 min intervals (36%) but fewer than those exposed to CCCP continuously (100%; Fig. 9h). Together, these data indicate that the PINK1:Parkin pathway is capable of integrating information about prior disturbances in mitochondrial activity at the level of mitochondrial Parkin recruitment and phospho-polyubiquitin deposition. Furthermore, these data also show that it is not necessary for PINK1 to be continuously associated with the OMM in order to promote Parkin and phospho-polyubiquitin accumulation and that this is a consequence of the difference in the rate of PINK1 and phospho-polyubiquitin degradation upon recovery of ΔΨm.

## Discussion

PINK1 and Parkin operate together as a molecular switch that is capable of responding to diverse mitochondrial insults and directing an appropriate response at both the level of individual mitochondria and the cell itself. They can stimulate apoptosis by inducing Parkin-dependent ubiquitination of the anti-apoptotic Bcl-2 family protein, Mcl-1, in response to catastrophic and irreparable mitochondrial damage or even salvage mitochondria damaged by ROS by directing the removal of oxidized mitochondrial proteins in mitochondria-derived vesicles, which are trafficked to lysosomes [40, 41]. Of all its functions, the ability of the PINK1:Parkin pathway to direct the autophagic removal of whole mitochondria exhibiting a complete and sustained loss of ΔΨm has received the most attention and study. Despite this, it still remains unclear how the pathway responds and makes coherent decisions about whether to keep or purge mitochondria in situations where ΔΨm is only partially lost or fluctuates over time, as might occur as part of the aging process or through exposure to low levels of mitochondrial stressors.

**Fig. 10:**
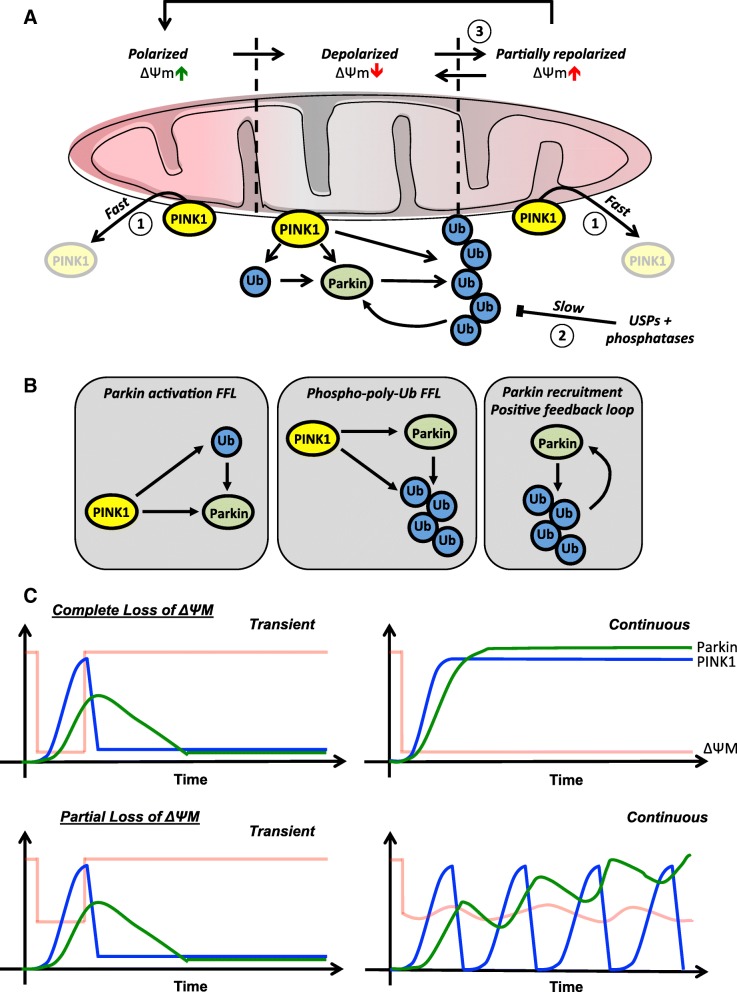
A model to describe the responses of the PINK1:Parkin pathway to mitochondrial stresses of differing magnitude and duration. **a** Model representing the changes in PINK1, Parkin, and phospho-ubiquitin chain levels at the mitochondria with changing ΔΨm. (1) In polarized, actively respiring mitochondria, PINK1 is rapidly imported, proteolytically cleaved, and degraded. (2) Phospho-ubiquitin chains are removed by mitochondria-associated USPs (and possibly phosphatases) in depolarized and repolarized mitochondria but the kinetics of this process are slow. (3) In partially depolarized mitochondria, small fluctuations in ΔΨm may generate short pulses of PINK1 activity. If these occur sufficiently frequently, ppUb chain density at the OMM may either be maintained or build due to their slow removal by USPs. **b** Diagrammatic representation of PINK1:Parkin pathway feedback loops. *Parkin activation FFL* – PINK1 activates Parkin directly through phosphorylation of the Parkin Ubl at Ser65 and indirectly by phosphorylating ubiquitin proteins at Ser65, which relieve autoinhibition of Parkin protein, constituting a coherent FFL. *Phospho-polyubiquitin FFL* - PINK1 activates Parkin directly through phosphorylation of the Parkin Ubl at Ser65, enabling Parkin to poly-ubiquitinate proteins on the OMM and these chains are phosphorylated by PINK1, forming a second coherent FFL. *Parkin recruitment positive feedback loop* – The ppUb chains produced by PINK1 and Parkin activity at the OMM serve as docking sites for the recruitment and activation of more Parkin. **c** Graphs to represent predicted changes in OMM-associated PINK1 (blue) and Parkin (green) levels in response to the indicated changes in ΔΨm (red)

Our current understanding of how the PINK1:Parkin pathway interprets changes in ΔΨm is informed by analysis of the pathway’s topology (Fig. 10a) and the feedback loops it incorporates (Fig. 10b). The two interlocking FFLs – the Parkin activation and the phospho-polyubiquitin FFLs – have been proposed to create a delay in the system such that ΔΨm must remain low for a sufficient length of time for initial phospho-polyubiquitin chains to assemble at the OMM. Once these have assembled, the Parkin recruitment positive feedback loop will generate a sufficiently high concentration of phospho-polyubiquitin chains at the OMM for autophagy receptors to be recruited [20]. In this way, the pathway exhibits a type of temporal gating and switch-like behaviour, removing only those mitochondria that exhibit a persistent loss of ΔΨm and sparing those that show only a transient loss. While this model is both compelling and useful, it assumes that the types of mitochondrial stress and damage that occur in vivo will result in simple binary changes in ΔΨm. Indeed, many if not most experiments that have probed the responses of the PINK1:Parkin pathway employ these binary changes by using high doses of CCCP to completely depolarize the mitochondria. Experimental data suggests that stress-induced changes in ΔΨm that occur in vivo are likely to be far more complex. For example, oxidative stress can trigger synchronous oscillations in ΔΨm in cardiac myocytes [42]. Even in unstressed cells, ΔΨm varies; it has been shown to fluctuate in neurons [29], flicker in a variety of cell types [43, 44], and even exhibit oscillations in pancreatic β-cells as a part of glucose-stimulated insulin secretion [27]. The magnitude of these variations can be large – up to 100 mV – with normal ΔΨm being approximately −150 mV in respiring mitochondria [29, 43, 45]. Quite how the PINK1:Parkin mitophagy pathway responds to and distinguishes between persistent partial loss of ΔΨm, stress-induced fluctuations in ΔΨm, or even normal changes in ΔΨm remain open questions.

As a first step towards answering these questions, we investigated how persistent partial loss of ΔΨm affected mitochondrial mass in cells treated with low and intermediate concentrations of the reversible oxidative phosphorylation inhibitor, CCCP. In SH-SY5Y cells, which have an intact PINK1:Parkin pathway, we found that even low doses of CCCP, which caused a slight but measurable decrease in ΔΨm, were capable of stimulating a loss of mitochondrial mass within 16 h, albeit less than that induced by CCCP doses that cause a complete loss of ΔΨm (Fig. 1h and i) – an apparent dose-dependent response to a reduction in ΔΨm. This could possibly be explained by a difference in the response between cells or the generation of heterogeneous populations of partially and fully depolarized mitochondria in individual cells at low and intermediate CCCP doses, as has previously been seen in cells exposed to CCCP for short periods of time [36], with only the mitochondria exhibiting the lowest ΔΨm being removed. In our experiments, where longer incubation periods allow CCCP to fully equilibrate within cells, it seems more likely that rather than creating mixed populations, the mitochondria will be more uniformly affected by the CCCP, a response supported by our TMRM staining experiments (Fig. 1a). It then becomes probable that the differences we see in mitochondrial mass after incubation with low and high CCCP doses are a product of differences in the kinetics of the PINK1 and Parkin response.

To investigate this possibility, we employed a live cell imaging approach, enabling us to measure the temporal dynamics of PINK1 and Parkin recruitment and loss at mitochondria in cells exposed to different CCCP treatment regimes. Consistent with previous studies showing stable accumulation of full-length endogenous PINK1 in cell populations incubated continuously with high doses of CCCP [36], we also observed stable accumulation of PINK1-EGFP in individual cells (Fig. 2b). However, in cells exposed to lower doses, PINK1 accumulation was transient, generally lasting less than 90 min (Fig. 2b and c). The reduction in PINK1 was sharp and asynchronous between cells and was found to occur concomitantly with a small increase in ΔΨm (Fig. 3a and b). Like voltage-gated channels, TIM23 undergoes a structural rearrangement, switching from an inactive to an active state if a specific threshold transmembrane electrical potential is exceeded [30]. Fluctuations in ΔΨm in partially depolarized mitochondria may therefore drive periodic bursts of TIM23 activity and inactivity as ΔΨm rises and falls around the threshold, producing corresponding pulses of mitochondrial PINK1 accumulation.

The rate of Parkin loss from repolarized mitochondria was markedly slower than that of PINK1 (Fig. 5). Our data suggests (although it does not directly prove) that this is a likely consequence of the slow removal of the ppUb on the OMM of mitochondria after repolarization. These chains have been shown to anchor Parkin at the mitochondria and also serve to recruit autophagy receptors like OPTN, which our data shows is also retained for many minutes after repolarization (Fig. 7). The persistence of ppUb chains is likely a consequence of the phosphorylation, which appears to partially protect polyubiquitin chains from the action of mitochondrially-targeted deubiquitinases including ubiquitin specific peptidase 8 (USP8), USP15, and USP30 [46], all of which have been shown to regulate Parkin-dependent mitophagy [47–50]. Our data suggests that it is the disparity in the timing of PINK1 degradation and ppUb removal that enables short but frequent bursts of PINK1 activity to drive incremental accumulation of Parkin and ppUb chains up to a threshold where autophagy receptors can be stably recruited. In this case, the threshold will be reached at a much slower rate than at completely depolarized mitochondria, where PINK1 is stably recruited. In this way the PINK1:Parkin pathway, like other prominent stress-responsive signalling mechanisms, may exhibit different dynamic behaviours in response to insults of differing magnitude [51], enabling the cell to expedite the removal of irretrievably damaged, non-functional mitochondria but also label, isolate, and temporarily defer the autophagy of partially depolarized mitochondria.

The importance of the phospho-polyubiquitin chain removal rate in shaping the dynamics of the response to mitochondrial insults raises the interesting possibility that the sensitivity of the PINK1:Parkin mitophagy pathway to recurrent changes in ΔΨm could be ‘tuned’ by adjusting the expression level of USPs. Potentially, this could also be achieved by altering the expression of autophagy receptors, including OPTN, which has been shown to be rate-limiting for mitophagy [39]. It could also hypothetically be controlled through the expression of phosphatases that dephosphorylate ppUb. This is likely to be important, given the various demands placed upon mitochondria in different cell and tissue types, and plausible, given that other stress-responsive pathways, such as p53, may show different dynamic behaviours in different cell types [52]. It is also possible that different mitochondrial quality control mechanisms, such as Nix and Bnip3, will be used in preference to PINK1:Parkin in cells that naturally produce large and repeating shifts in their activity to avoid inappropriate mitophagy [53].

Finally, one potential caveat that was raised during peer review of this manuscript was the effect of FBS on the ability of CCCP to induce mitochondrial depolarization. As shown by Soutar et al, FBS reduces the potency of CCCP such that concentrations of 10 μM or greater are required to completely depolarize mitochondria in cells cultured in the presence of 10% FBS whereas concentrations < 1 μM will achieve comparable effects in the absence of FBS [54]. As all experiments were performed in the presence of 10% FBS, we do not believe that this observation impacts the findings of our study in any way. In many of our experiments, ΔΨm (measured using TMRM) and the localization of fluorescently labelled mitophagy regulators was monitored in the same living cells. We believe that our work and the data presented in Soutar et al emphasize the importance of using consistent cell growth conditions in studies using CCCP that seek to investigate links between changes in ΔΨm and the activity and dynamics of mitophagy regulators.

## Conclusions

Our data shows differences in the temporal dynamics of PINK1 and Parkin accumulation and loss at mitochondria exposed to different concentrations of the protonophore, CCCP, with intermediate levels of mitochondrial depolarization giving rise to bursts of PINK1 activity caused by small fluctuations in ΔΨm. By artificially inducing these PINK1 pulses through controlled titration of CCCP, we also show that the pathway can integrate temporally separated periods of mitochondrial depolarization through incremental accumulation of Parkin and ppUb. We propose that this provides a mechanism for cells to discriminate between mitochondria exhibiting different degrees of impairment and control the rate at which they are autophagized. Importantly, it also demonstrates that stable association of PINK1 with the OMM is not essential for mitophagy and likely only occurs at the most heavily damaged mitochondria.

## Methods

### Cell culture, plasmids, and transfections

HeLa (CCL-2) human cervical adenocarcinoma cells and SH-SY5Y (CRL-2266) human neuroblastoma cells were obtained from ATCC (Manassas, VA). HeLa cells were cultured in DMEM supplemented with 10% FBS, 200 mM L-glutamine, and 100 units/mL penicillin and streptomycin. SH-SY5Y cells were cultured in a 1:1 mix of Eagle’s minimum essential medium and F12 Medium supplemented with 10% FBS and 100 units/mL penicillin and streptomycin. Both cell lines were maintained at 37 °C in a humidified 5% CO_2_ atmosphere. Cells were seeded in either 60 mm tissue culture treated dishes at a density of 5 × 10^6^ cells/dish for biochemical analysis or 35 mm glass-bottomed dishes (Celvis) at a density of 1 × 10^5^ cells/dish for live cell microscopy. All experiments were performed in the appropriate cell growth medium supplemented with 10% FBS.

The pMito-mCherry plasmid construct was produced by cloning mCherry cDNA into the pShooter-MITO vector (Life Technologies, USA), in-frame with the mitochondrial targeting presequence of subunit VIII of human cytochrome c oxidase. pEYFP-Parkin was produced by cloning the wild type, full-length human Parkin cDNA sequence into pEYFP-C1 (Takara, USA), in frame with EYFP. pmCherry-Parkin was subsequently produced by replacing the EYFP coding sequence with the mCherry sequence. pPINK1-EGFP was produced by cloning the wild type, full-length human PINK1 cDNA sequence into pEGFP-N1 (Takara, USA), in frame with EGFP. The pOPTN-EGFP (#27052) expression vector was purchased from Addgene. Cells were transfected with the indicated plasmid constructs using a linear polyethylenimine-based transfection reagent (Polyscience, USA) 24 h after cells were plated.

### Antibodies

The primary antibodies used for immunofluorescent staining and immunoblotting-based experiments were as follows: actin (A2066, Sigma), DNA (61,014, Progen Biotechnik GmbH), K48 polyubiquitin (05–1307, EMD Millipore), K63 polyubiquitin (Apu3, 05–1308, EMD Millipore), mono- and polyubiquitinated conjugates monoclonal antibody (FK2, Enzo Lifesciences), Parkin (ab15954, Abcam), phospho-ubiquitin Ser65 (#37642; Cell Signaling), PINK1 (D8G3, Cell Signaling), and TOM20 (D8T4N, Cell Signaling). Detection of primary antibody-binding was performed using anti-mouse Alexa Fluor 555 conjugated antibodies (Life Technologies) or anti-rabbit Alexa Fluor 647 conjugated antibodies (Life Technologies) for immunofluorescent staining experiments and HRP-conjugated anti-rabbit or anti-mouse secondary antibodies (Santa Cruz Biotechnology), as appropriate, for immunoblotting.

### Immunoblotting

Cells for immunoblotting were lysed in RIPA buffer containing both protease and phosphatase inhibitor cocktail. The protein concentration of the samples was determined by BCA assay. After normalizing protein concentrations, the samples were boiled in Laemmli buffer and the proteins resolved by SDS-PAGE prior to western blotting for the indicated proteins.

### Time lapse microscopy and immunofluorescence

Live cells were imaged using a Nikon Ti-Eclipse wide field microscope equipped with a CFI Plan 40x oil immersion NA 1.30 objective, Intensilight epifluorescence illuminator, computer-controlled stage (Nikon), CoolSNAP MYO camera (Photometrics), and a full environmental enclosure with CO_2_, humidity, and temperature control (InVivo Scientific). The microscope was controlled using Elements software (Nikon). Images were acquired at the indicated intervals (typically 3 min). EGFP and EYFP was excited through a 465–495 nm excitation filter and emitted light was detected through a 515–55 nm barrier filter reflected from a 505 nm dichroic mirror. TMRM and mCherry fluorescence was excited through a 535–550 nm excitation filter and emitted light was detected through a 610–675 nm barrier filter reflected from a 565 nm dichroic mirror. For experiments involving the measurement of ΔΨm, cells were incubated with 10 nM TMRM for 15 min at 37 °C prior to imaging. TMRM concentration was kept constant even if CCCP concentration was varied during the experiment.

The live cell experiments depicted in Fig. 7 and the imaging of all immunofluorescently stained samples was conducted using a Zeiss LSM700 confocal laser scanning microscope equipped with a Definite Focus II autofocus system, an Incubator XL S1 full environmental enclosure with CO_2_, humidity, and temperature control, and a Plan-Apochromat 63x NA 1.4 oil immersion DIC M27 objective (Carl Zeiss). Alexa Fluor 555 and mCherry fluorescence was excited using a 555 nm laser and emitted light was detected through a LP560 filter. Alexa Fluor 647 fluorescence was excited using a 639 nm laser and emitted light was detected through a LP640 filter. EGFP and EYFP fluorescence was excited using a 488 nm laser and emitted light was detected through a SP555 filter. DAPI (NucBlue™ Fixed Cell ReadyProbes™ Reagent; ThermoFisher Scientific) fluorescence was excited using a 405 nm laser.

### Image analysis

For the live cell microscopy experiments conducted on the Nikon Ti-Eclipse, 14-bit Nikon nd2 timecourse image files were acquired using Elements software (Nikon) and analysed using Fiji [55]. For each recorded fluorescence channel, the images were thresholded to remove background signal. For experiments involving PINK1-EGFP or TMRM, the fold change in fluorescence throughout the entire mitochondrial network was measured at each time point in individual cells. For experiments involving EYFP-Parkin, the fold change in EYFP fluorescence was measured in a representative region of the mitochondrial network at each time point. Line scan measurements were made using the intensity profile tool in Elements. For the live cell microscopy experiments conducted on the Zeiss LSM700, 16-bit image files were acquired using Zen software (Zeiss). Pearson’s correlation coefficient and line scan intensity profile measurements were also made using the same software. For the experiments described in Fig. 1e-f, which involved the immunofluorescent staining of mtDNA nucleoids, we utilized an anti-DNA antibody that has been previously shown to preferentially stain mtDNA [37, 56]. The number of mtDNA puncta was quantified for 20 cells per condition. Vehicle treated control cells contained an average of 76 (±24) puncta per cell. Overall, > 3000 puncta were counted across 60 cells.

### Statistical analysis

Statistical analyses were performed in GraphPad Prism 7 (GraphPad, USA) using the tests indicated in the figure legends.

## Additional files

Additional file 1: **Figures S1.** Full-length PINK1-EGFP is stabilized in HeLa cells post-CCCP treatment. PINK1-EGFP expressing HeLa cells and untransfected control cells were incubated for the indicated times with 10 μM CCCP prior to lysis and western blot analysis for PINK1 and actin proteins. Full-length exogenous PINK1-EGFP fusion proteins were stabilized in CCCP-treated cells, showing a similar fold increase to endogenous PINK1 proteins. (PDF 315 kb)

Additional file 2: **Figures S2.** EYFP-Parkin remains colocalized with poly-ubiquitin at the mitochondria for at least 2 h after CCCP wash-out. HeLa cells expressing EYFP-Parkin (green) were treated with a 60 min pulse of 10 μM CCCP, fixed at the indicated timepoints and stained with DAPI (blue) and the pan-ubiquitin antibody, FK2, which detects both mono- and polyubiquitinated proteins (red) then imaged by confocal microscopy. Scale bars represent 50 μm. (PDF 7121 kb)

Additional file 3: **Figure S3.** Quantification of Parkin+OPTN-positive mitochondria after CCCP washout. HeLa cells expressing mCherry-Parkin and OPTN-EGFP were imaged by live cell microscopy and were either pulsed for 60 min or treated continuously with 10 μM CCCP. The number of mitochondria positive for *(A)* mCherry-Parkin or *(B)* mCherry-Parkin and OPTN-EGFP was quantified at 120 min after initial treatment. Data is from 3 biological repeats with a minimum of 19 cells per condition. Statistical differences between the two conditions were appraised using a two-tailed, unpaired *t*-test. Statistical significance is indicated as follows: *, *p* < 0.05. (PDF 55 kb)

## Data Availability

All data generated or analysed during this study are included in this published article. All plasmid constructs used in this study are available on request from David E. Nelson.

## Abbreviations

CCCP: Carbonyl cyanide *m*–chlorophenyl hydrazine
FFL: Feed-forward loop
NDP52: Nuclear dot protein 52
NF-κB: Nuclear factor-kappa B
OMM: Outer mitochondrial membrane
OPTN: Optineurin
PINK1: PTEN-induced putative kinase 1
TOM:TIM: Translocase of outer membrane:translocase of inner membrane
Ubl: Ubiquitin-like domain
USP: Ubiquitin specific peptidase
ΔΨm: Mitochondrial membrane potential
