# Supplementary figures and images for: Temporal integration of mitochondrial stress signals by the PINK1:Parkin pathway
Source: BMC Mol Cell Biol. 2019 Aug 14;20:33. doi: 10.1186/s12860-019-0220-5 (PMC6694515; doi:10.1186/s12860-019-0220-5)

Endogenous PINK1

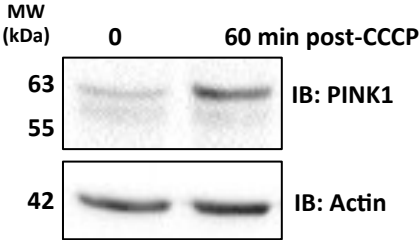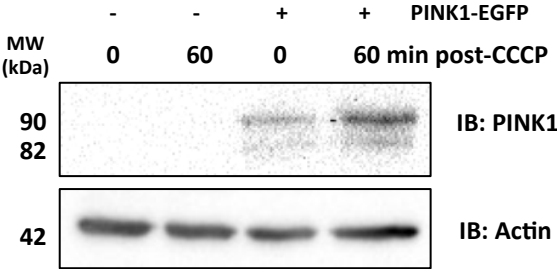

Supplement: Supplementary file 1 — Figures S1. Full-length PINK1-EGFP is stabilized in HeLa cells post-CCCP treatment. PINK1-EGFP expressing HeLa cells and untransfected control cells were incubated for the indicated times with 10 μM CCCP prior to lysis and western blot analysis for PINK1 and actin proteins. Full-length exogenous PINK1-EGFP fusion proteins were stabilized in CCCP-treated cells, showing a similar fold increase to endogenous PINK1 proteins. (PDF 315 kb) [file 12860_2019_220_MOESM1_ESM.pdf]

10  $\mu$ M CCCP – 60 min Pulse

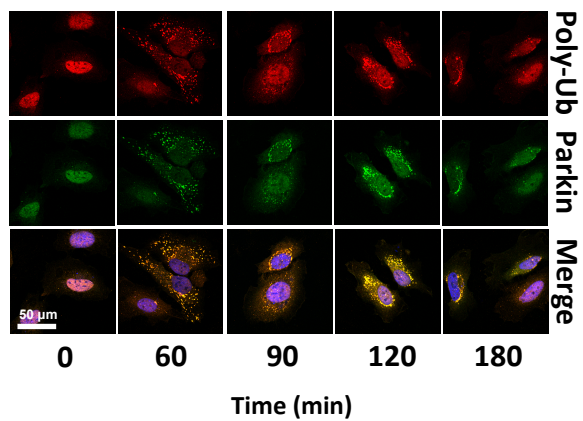

Supplement: Supplementary file 2 — Figures S2. EYFP-Parkin remains colocalized with poly-ubiquitin at the mitochondria for at least 2 h after CCCP wash-out. HeLa cells expressing EYFP-Parkin (green) were treated with a 60 min pulse of 10 μM CCCP, fixed at the indicated timepoints and stained with DAPI (blue) and the pan-ubiquitin antibody, FK2, which detects both mono- and polyubiquitinated proteins (red) then imaged by confocal microscopy. Scale bars represent 50 μm. (PDF 7121 kb) [file 12860_2019_220_MOESM2_ESM.pdf]

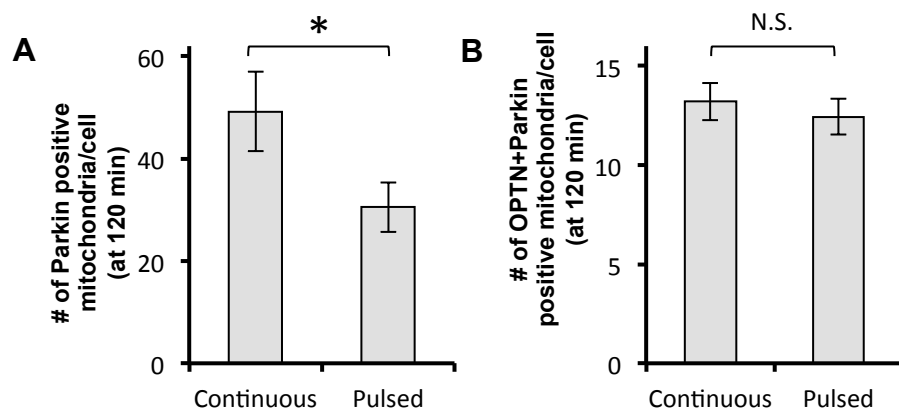

Supplement: Supplementary file 3 — Figure S3. Quantification of Parkin+OPTN-positive mitochondria after CCCP washout. HeLa cells expressing mCherry-Parkin and OPTN-EGFP were imaged by live cell microscopy and were either pulsed for 60 min or treated continuously with 10 μM CCCP. The number of mitochondria positive for (A) mCherry-Parkin or (B) mCherry-Parkin and OPTN-EGFP was quantified at 120 min after initial treatment. Data is from 3 biological repeats with a minimum of 19 cells per condition. Statistical differences between the two conditions were appraised using a two-tailed, unpaired t-test. Statistical significance is indicated as follows: *, p < 0.05. (PDF 55 kb) [file 12860_2019_220_MOESM3_ESM.pdf]
